# A Quantitative Study of the Impact of Functional Classification on Competitive Anxiety and Performance Among Wheelchair Basketball Athletes

**DOI:** 10.3389/fpsyg.2020.558123

**Published:** 2020-09-23

**Authors:** Natasja Bosma, Nico W. Van Yperen

**Affiliations:** Department of Psychology, University of Groningen, Groningen, Netherlands

**Keywords:** adaptive sports, Paralympic sports, Para sports, disability, impairment, WAI-S, CSAI-2, pre-competitive appraisal

## Abstract

In adaptive sports (also known as Para sports, disability sports, or Paralympic sports), athletes are assigned to classes that indicate their functional potential, regardless of talent, training, or experience. The aim of the present study among wheelchair basketball athletes (*n* = 141) was to explore the role of functional classification as a potential stressor. Specifically, we looked into the anecdotal relationship between classification and athletes’ concern about “performing in accordance with one’s class.” Based on a serial mediation research model, we examined the links between functional classification and three outcome variables (i.e., cognitive worry, somatic arousal, and game performance) through the mediator variables of perceived competitive demands and sport-specific self-efficacy. Unexpectedly, we did not find any evidence of a classification effect on either the mediator variables or competitive anxiety. However, we did find positive correlations between functional classification and athletes’ contribution to their team’s score, which align with research supporting the proportionality and the validity of the functional classification system. Moreover, regardless of classification, mediation analyses revealed an indirect link between perceived competitive demands and cognitive worry through sport-specific self-efficacy. These findings suggest that, regardless of classification, athletes’ self-efficacy may be increased by managing their appraisals of competitive demands and that their cognitive worries may be reduced by self-efficacy interventions.

## Introduction

Classification is a well-known concept in the world of sports in general, and sports played by persons with a disability in particular. Its main purpose is to promote participation by reducing the likelihood of one-sided competition (Tweedy and Vanlandewijck, 2011). In order to achieve this aim, classification systems are designed to group athletes according to a unit of classification so as to minimize the impact of that unit on the outcome of competition. This approach is taken, for example, to separate male from female athletes, or to create weight classes or age groups. As the respective units of classification in these examples—sex, weight, and age—are obvious determinants of performance, their impact is taken out of the competition. Sex, weight, and age classification are examples of selective classification, in which determinants of performance are the dividing unit, while training and talent are not considered. Alternatively, the dividing unit can be performance itself, giving rise to performance classification. The handicap system used in amateur golf is an example of this form of classification: an amateur golfer’s playing handicap is based on their previous performances, which means that they can influence their classification through effective training and performing well on the course (Tweedy and Vanlandewijck, 2011).

Classification plays a fundamental role in adaptive sports, which are also referred to as Para sports, disability sports, or Paralympic sports. In this paper, we will use the term adaptive sports throughout. Classifying athletes with diverse disabilities—and, therewith, diverse degrees of impairment—enables them to compete with and against each other in the same sport in an equal manner. As for any classification system, the purpose is to promote equal participation in sports (International Paralympic Committee, 2015a). To create such equality, the impact of an athlete’s impairment on the outcome of competition needs to be minimized (Tweedy et al., 2014). Thus, the unit of classification is the level of impairment that an athlete brings to the game (i.e., selective classification). As a result, classification systems in adaptive sports are far more complex than in conventional sports due to the great variability in this unit of classification. Athletes have different disabilities, and even among athletes with the same disability there is great variance in the resulting impairment.

The International Paralympic Committee (IPC) prescribes two essential requirements for classification systems in adaptive sports. First, they need to set criteria for who is eligible to compete in the sport in question, which means they have to set guidelines for the types of impairment that are eligible and to define what the minimum severity of these eligible impairments must be (i.e., minimum impairment criteria). Second, they have to define sport classes that represent different levels of impairment and provide guidelines for allocating those sport classes to athletes with eligible impairments based on the extent to which they are able to execute sport-specific tasks and activities. The IPC further mandates that classification systems are evidence-based, meaning that they should be supported by solid research (Tweedy and Vanlandewijck, 2011; Tweedy et al., 2014; International Paralympic Committee, 2015a). Most, if not all, classification systems in adaptive sports are designed in alignment with these IPC guidelines, policies, and procedures. Classification systems vary with regard to the number of classes and whether function (impairment level) is assessed with on-court observation, off-court tests, or a combination of both (International Paralympic Committee, 2015b, 2016; Van der Slikke et al., 2020).

### Classification in Wheelchair Basketball

Wheelchair basketball has been one of the most popular adaptive sports from the moment it started to develop at the end of the Second World War (Labanowich and Thiboutot, 2011). The eligible population for this sport has become more heterogeneous over the years as the philosophy behind athlete classification has evolved. Today, wheelchair basketball athletes are individuals with diverse disabilities, the most common ones being spinal cord injuries, lower limb deficiencies (due to amputation or illness), poliomyelitis, spina bifida, and cerebral palsy. To compare these athletes and allocate them to classes for competition, the functional classification system advocated by Strohkendl (1978, 1996, 2001) is still considered a “satisfactory” method from the participants’ perspective (i.e., athletes, coaches, and classifiers; Molik et al., 2017). In this system, an athlete’s functional potential is determined by the range, strength, and coordination of trunk function (volume of action), lower limb function, and upper limb function. Athletes’ levels of impairment of these body functions affect their abilities to execute movements and skills that are relevant to the game of wheelchair basketball: pushing, pivoting, shooting, rebounding, dribbling, passing, and catching.

The functional classification system in wheelchair basketball distinguishes between eight classes, ranging from 1.0 to 4.5, with higher numbers indicating greater functional potential. If we look at the volume of action, for example, we see that athletes in the 1.0 class have no controlled trunk movement in any direction whatsoever, whereas athletes in the 2.0 class do have partially controlled trunk movement, but only in the forward and vertical (transvers) direction. Athletes in the 3.0 class have controlled and full trunk movement in both the forward and vertical plane, but they lack that movement sideways, whereas athletes in the 4.0 class have full trunk movement in almost all directions (significantly weak trunk movement in one sideways direction). This distinguishes them from athletes in the minimal disability class, who have no limitations in their trunk movement at all. The intermediate classes—1.5, 2.5, and 3.5—are reserved for athletes who are between two classes because they have characteristics of both classes (International Wheelchair Basketball Federation, 2014).

To be assigned a classification, athletes are observed during one or more games by classifiers, who are trained to recognize the unique features of a functional class in athletes’ play. The functional potential of a team consists of the sum of classification points of the five players on the court, and at any given time in a game, this sum of points must not exceed 14.0. In this way the functional potential of teams is equalized and the result of competition is directly related to performance and not to disability.

### Psychological Aspects of the Classification System

The fundamental role of classification in adaptive sports, as highlighted above, also makes classification a potential source of stress for the participating athletes (Hanrahan, 2005; Howe, 2008). Martin (2018), for example, refers to the evaluative process that might elicit a stress response, but also to the looming possibility of being assigned a higher (functionally stronger) class in the case of reclassification, which would result in having to compete against athletes who perform at a higher level. In team sports, like wheelchair basketball, the restriction regarding the maximum team functional potential on court (14.0) adds an additional layer to classification as a potential stressor because the classification or reclassification of an individual team member can affect team strategy and the division of playing time within the team (Martin, 2018). To illustrate this, consider a starting lineup composed of five players with the classifications of 4.5, 3.5, 3.0, 2.0, and 1.0. If the 2.0 athlete in this lineup is reclassified as 2.5, the coach either has to substitute this athlete for a bench player with classification of 2.0 or lower or substitute one of the other four athletes to stay within the maximum sum of 14.0 points. This implies that, in a team context, functional classification may promote individual concerns about lack of court time while playing well, being on the bench while feeling one could contribute, or being left on the court while playing poorly; these are all among the “on-court concerns” that were specified as sources of stress among wheelchair basketball athletes by Campbell and Jones (2002).

**FIGURE 1:**
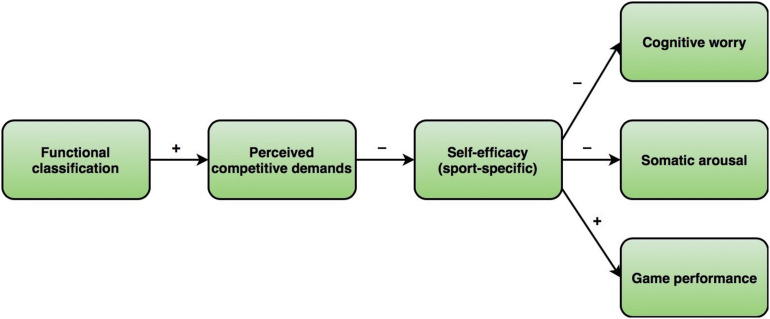
Conceptual research model, with plus and minus signaling the direction of hypothesized relationships.

Another classification-related potential source of stress is concern about performing in accordance with one’s functional class. Among insiders, there is anecdotal (i.e., non-documented) evidence that athletes sometimes feel they have to outperform team members with lower classifications to warrant their position in the lineup. The relevance of this type of performance expectation is signaled by the existence of intentional misrepresentation, or “sandbagging,” which refers to the phenomenon that athletes exaggerate their impairment by deliberately underperforming during the classification process (Howe, 2008; Lindemann, 2008; Tweedy et al., 2014; Martin, 2018). In this way, they hope to gain an unfair advantage, by being placed in a lower functional class. Thus, functional classification has the potential to color athletes’ performance expectations. Hence, we assumed sequential links between functional classification and athletes’ perceived competitive demands, sport-specific self-efficacy, competitive anxiety, and sports performance, which are summarized in Figure 1 and discussed in more detail in the next paragraphs.

#### Perceived Competitive Demands

Performance expectations are among the most common stressors experienced by athletes (Sarkar and Fletcher, 2014). For wheelchair basketball athletes, a relevant factor in this regard may be the (self-imposed or other-imposed) expectation that athletes should perform in accordance with their functional class. The classification system follows an ordinal scale, from 1.0 to 4.5 in 0.5-point steps, which can give rise to the expectation that the (individual) field performance of athletes should follow the same proportional pattern. This would imply that athletes with higher classifications are expected to demonstrate a stronger (individual) field performance because they have greater functional potential. Yet, the available empirical evidence is ambiguous about the proportionality between the functional classes and athletes’ functional potential, and whether differences in functional potential are reflected in performance.

Most research into the functional classification system of wheelchair basketball has been focused on validating the system, by exploring whether the different levels of functional classification are reflected in physiological measures (aerobic and anaerobic performance), biomechanics (motion and force characteristics), and sports performance. In the latter category, there are multiple studies that have explored links between classification and the performance of wheelchair basketball-specific skills and techniques, including wheelchair propulsion (speed, agility, endurance), passing (accuracy, explosiveness), ball handling, and shooting (mechanics, accuracy). These studies typically showed no differences between the midpoint (2.0–3.0) and the highpoint (4.0/4.5) classes (Brasile, 1986, 1990; Vanlandewijck et al., 1995; Brasile and Hedrick, 1996; Molik and Kosmol, 2001; Malone et al., 2002; Doyle et al., 2004; De Groot et al., 2012; Cavedon et al., 2015; Gil et al., 2015; Molik et al., 2017; Saltan and Ankarali, 2017). In contrast, studies relying on game analyses demonstrate clear performance differences between classification groups, particularly in athletes’ abilities to execute more dynamic elements, such as blocks, rebounds, and turnovers (Molik and Kosmol, 2001; Van der Slikke et al., 2018).

This raises the question as to why differences in performance between the functional classes do appear when game performance is analyzed, but not when test batteries aiming to represent that game performance are used. Therefore, De Groot et al. (2012) examined the validity of commonly used skills and techniques tests in test batteries to assess wheelchair basketball performance. Their results suggest that although tests aim to measure diverse aspects of performance, they typically only measure two underlying constructs: shooting and speed. These are both elements of the game in which an athlete’s volume of action is less influential than in the more dynamic aspects (Molik and Kosmol, 2001), including penetrating the offensive zone, rebounding, and stealing the ball. Therefore, differences in functional potential may be more apparent in dynamic game settings than in test settings. Recently, Bergkamp et al. (2019) also concluded that samples of relevant sports behavior (i.e., performance in small-sided soccer games) have more fidelity than isolated skills tests or “signs,” and such samples are therefore more useful in identifying and predicting game performance.

Vanlandewijck et al. (2003) used game performance to examine whether the functional classes are in the correct proportion relative to each other. They found that, overall, the proportionality of the system represents the functional potential of the athletes, but they did note a slight underestimation of the functional potential of athletes in the 2.0 and 3.0 classes (cf. Vanlandewijck et al., 2004). These findings align with the results of a recent study conducted by Francis et al. (2019), who analyzed game performance to identify the key dynamic variables associated with team success in elite men’s wheelchair basketball. They found that the odds of winning increased when lineups were composed of athletes from the midpoint classes (2.0–3.5) rather than from the extreme classes (1.0–1.5 and 4.0–4.5).

Overall, the results of these studies imply that the (individual) field performance demonstrated by “expensive” highpoint athletes is not as strong as suggested by the ordinal scale of the functional classification system. Note that these athletes account for a majority of the team’s functional potential on the court (14.0 points in total), thus limiting the functional potential that can be contributed by the other players. Therefore, we anticipated a positive relationship between classification and athletes’ perception of competitive demands (see Figure 1).

#### Sport-Specific Self-Efficacy

Self-efficacy beliefs refer to what individuals believe they are capable of doing under specific circumstances (Bandura, 1997). Contrary to more global and stable forms of self-concept, like self-esteem or self-confidence, self-efficacy beliefs may fluctuate depending on the domain, the situation, or even the specific task at hand (Bandura, 1997; Marsh et al., 2017). According to Bandura (1997), self-efficacy beliefs are shaped by information from four major sources: (1) mastery experiences in the form of successful performance accomplishments, (2) vicarious experiences through social comparison and observational learning, (3) verbal persuasion through self-talk and feedback of relevant others, and (4) physiological and affective states. Based on these antecedents, Rigotti et al. (2018) examined the role of exceeding demands on career-related self-efficacy. They found that demands exceeding one’s own capacities have a negative impact on self-efficacy beliefs, not only because these demands endanger performance accomplishments, but also because the stress of high demands causes emotional strain. When we apply this to the present wheelchair basketball context, it suggests that the appraisal of classification-related competitive demands may influence athletes’ sport-specific self-efficacy beliefs. Specifically, when perceived competitive demands are high and do not fit athletes’ perception of their ability to meet them, the resulting emotional strain may negatively affect the athletes’ sport-specific self-efficacy. Hence, we predicted a negative relationship between perceived competitive demands and athletes’ sport-specific self-efficacy. As shown in Figure 1, we expected sport-specific self-efficacy, in turn, to be related to competitive anxiety and game performance (Moritz et al., 2000; Feltz et al., 2008).

#### Competitive Anxiety and Game Performance

Competitive anxiety is a multidimensional concept that refers to an athlete’s negative cognitive and somatic responses to perceived competitive demands. The cognitive dimension refers to the worrying thoughts and concerns that an athlete may have in anticipation of the upcoming event, such as concern about performing poorly or being evaluated negatively by others (Martens et al., 1990). The somatic dimension refers to indications of physiological arousal that an athlete may experience, such as rapid breathing, an increased heart rate, or a tense stomach. Although the two dimensions of competitive anxiety are conceptually different (Nitschke et al., 2001), they are interrelated and have the tendency to trigger and strengthen each other (Martens et al., 1990; Beilock et al., 2017; Moran and Toner, 2017).

The negative relationship between self-efficacy and pre-competitive anxiety is well-established in research among able-bodied athletes, indicating that self-efficacious athletes experience less cognitive and somatic anxiety prior to competition (Feltz and Lirgg, 2001; Feltz et al., 2008). Although Schliermann and Stoll (2007) did not find this link among female wheelchair basketball athletes, this may be because their study was focused on trait anxiety rather than state anxiety. Moreover, they assessed self-efficacy and trait anxiety in the rest period between days of competition of a 2-day event rather than in a relatively short time frame before competition.

Furthermore, a meta-analysis of studies among able-bodied athletes clearly revealed a positive relationship between self-efficacy and sports performance (Moritz et al., 2000). This finding suggests that athletes with a strong sense of self-efficacy perform better because they, for example, select more challenging goals, are more optimistic about the outcome of these goals, invest more time in the pursuit of their goals, and persist longer in their efforts (Feltz et al., 2008; Zimmerman et al., 2017). Similar results have been observed in research among athletes in adaptive sports (for an overview, see Martin, 2018). For example, Katartzi et al. (2007) found that wheelchair basketball athletes’ self-efficacy was predictive of their performance in a passing task. Similarly, among wheelchair road racers, Martin (2002) found that racers who were more self-efficacious performed better than less self-efficacious racers.

To summarize, our aim was to add to the extant knowledge and understanding of the functional classification system in wheelchair basketball by exploring its role as a competitive stressor. The hypothesized relationships are displayed in Figure 1, which shows that we predicted that there are links between functional classification and both competitive anxiety and game performance through perceived competitive demands and sport-specific self-efficacy.

## Materials and Methods

### Study Design, Setting, and Participants

A quantitative correlational study was designed to test whether, and to what extent, relationships exist among the different variables in our research model (see Figure 1). The participants of the study consisted of wheelchair basketball athletes from teams that competed in the German state of North Rhine-Westphalia (NRW), which is one of the five regions of the German wheelchair basketball competition. This competition contains five competitive levels. The first level is the 1e Bundesliga, which is a nationwide league; the 2e Bundesliga, the second level, is divided into a northern and a southern league. The subsequent three levels are denoted as the Regionalliga, the Oberliga, and the Landesliga, respectively. The lowest league, the Landesliga, was *a priori* omitted, as relatively more inexperienced players and youth players are present in this league than in the higher leagues. Most of these players have limited or no awareness of the principles of the classification system, nor of their own classification.

The wheelchair basketball competition in Germany is a mixed competition in which both male and female athletes play on the same teams. Teams also regularly include both youth and adult players, as well as athletes of diverse levels of experience. On a national level, the functional classification system is therefore expanded with “bonus points” to integrate the classification of sex, age, and experience within the same system. Specifically, teams are allowed to use a larger sum of classification points when the five players on the court include a female athlete (1.5 additional points), an under-18 athlete (1.0 additional point), or an athlete with less than 2 years of experience (1.0 additional point). These bonus points are not specific to the German leagues; they are, for example, also used in mixed Euroleague tournaments.

It is important to note that coaches can combine the bonus points within certain limits: across all five German leagues, the maximum bonus for each male and female athlete is 1.0 and 2.0, respectively. In the two Bundesliga leagues, the maximum bonus points per team on the court (i.e., five athletes) are, additionally, limited to 3.0 and bonus points for inexperienced players do not apply. Furthermore, across all five leagues, the maximum number of classification points per team on the court (bonus points excluded) has been increased by half a point to 14.5 (Fachbereich Rollstuhlbasketball des DRS, 2017).

Both purposive and convenience sampling methods were used in the selection of participants. Athletes were not selected individually, but based on their membership within a particular team. First, a suitable measurement context had to be chosen to measure meaningful and valid states of competitive anxiety. For example, a low-stakes context, in which the game result is already obvious in advance, would not result in a realistic impression of an athlete’s cognitive worry and somatic arousal. Therefore, the study was conducted in the second half of the competitive season, so that potentially close games could be selected from the playing schedules based on two criteria: (1) the mid-season ranking of the teams, and (2) the game results of match-ups in the first half of the season. These criteria were combined because teams that followed each other in ranking were sometimes still separated by a large gap in competition points and goal difference, making a close game less likely. The results of match-ups in the first half of the season were thus consulted to identify—for each team—the other teams close in mid-season ranking that would most likely be the best opponent for a potentially close game.

From each of the four leagues considered, as many potentially close games as possible were selected, so as to include a maximum number of wheelchair basketball athletes in the sample. In this way, the sample’s potential to be generalized to a larger wheelchair basketball population was maximized. There were only a few athletes who did not participate in the study, despite being approached to do so. The most common reason for this was related to language limitations; not all of the athletes were native Germans and some of them were not sufficiently fluent in the German language to be able to understand what was asked of them. Time constraints were another reason; some athletes were late for their game and had no time left to fill out the questionnaire. There were only two athletes who refused to participate without specifying their reasons for doing so.

The final sample consisted of 141 wheelchair basketball athletes, representing 20 teams that competed in the NRW region. As can be seen in Table 1, which provides a demographic profile of the study sample, the participants are not equally distributed over the four competitive levels; a majority of the participants (80.1%) were active in the (more recreational) Regionalliga and Oberliga. There is no overlap between the subsamples from the different competitive levels; athletes who were active in more than one league (i.e., regular player in one team and reserve player in the other), and who were involved in more than one of the selected games, were asked to participate in the study during the game of the team in which they were a regular player.

**TABLE 1:** Demographic characteristics of the study sample.

Variables	Category	*N*	*n*	%
Competition level	1e Bundesliga	141	12	8.5
	2e Bundesliga		16	11.3
	Regionalliga		49	34.8
	Oberliga		64	45.4
Sex	Male	141	116	82.3
	Female		25	17.7
Age	≤18 years	141	9	6.4
	>18 years		132	93.6
Experience	<2 seasons	141	9	6.4
	≥2 seasons		132	93.6
Classification	1.0–1.5	141	32	22.7
	2.0–2.5		28	19.9
	3.0–3.5		26	18.5
	4.0–4.5		55	39.0

### Procedure

Teams that were involved in the selected games were contacted a week before the scheduled game date through their team contact person who was registered with the AG-RBB Nordrhein-Westfalen. These contact persons received an e-mail with an explanation of the context in which the study was conducted, the question whether the athletes of the team would be willing to participate in the study before and after the next game, the time investment that was required from the athletes, and the assurance that they could contact the researcher at any time if questions would arise.

On the day of competition, the coach of the selected team was approached as soon as he or she arrived at the location, to make sure that no misunderstandings had occurred. A single-page pre-game questionnaire was assembled, with demographic questions and a measure of perceived competitive demands on the front side and measures of sport-specific self-efficacy and competitive anxiety on the back side. This pre-game questionnaire was administered 30–40 min prior to the scheduled start of the game, which is considered to be the most optimal moment for assessing competitive state anxiety (Craft et al., 2003). Hence, the administration of the questionnaire would not disturb the regular warm-up activities of the team and the athletes would not be distracted from the questions by the imminent start of the competition. A post-game questionnaire to measure athlete performance was administered to the coach of the team 5 to 10 min after the game had ended.

All questionnaires were printed on heavy paper (200 g), so that athletes could fill them out themselves while sitting in their sports wheelchair, without needing any clipboard or other hard surface to write on. The questionnaires were group-administered because they were administered to all the athletes from the same team who agreed to participate in the study at once. This setup comes with an inherent risk of conformity, meaning that athletes may have felt pressured to participate in the study because all their teammates did so. To enable us to link the questionnaires with objective performance indicators (by name) collected at the end of the season, the participants were asked to write their names on the questionnaires. They were informed that their answers would be treated confidentially and that the data would be anonymized as soon as the data files were combined. The participants’ choice to return the completed questionnaires implied informed consent to participate. Notwithstanding the procedural limitations, non-participation in the research was minimal.

### Measures

#### Functional Classification

The participating athletes were asked to indicate (1) their functional class and (2) whether they qualify for any bonus points regarding sex, age, or experience by ticking the appropriate boxes of the multiple-choice items. The latter bonus-related demographic characteristics were assessed because bonus points may have an impact on an athlete’s classification-related perceived competitive demands and, accordingly, should be statistically controlled for in our analyses. For example, when a team on the court has reached its maximum number of classifications points (14.5 in Germany), a 2.5 male adult player can be legally substituted for a 3.5 male under-18 player. Although the 1.0 point bonus is not subtracted from this particular under-18 player’s individual classification, he may be compared (by himself or others) to a 2.5 player.

#### Perceived Competitive Demands

To be able to capture the competitive demands as they are perceived and experienced by the athletes themselves, a single-item self-report measure was developed. The participants were asked to indicate on a five-point icon-based Likert scale how they thought their performance compared to the expectations people have about players with their classification. The icons reflected answers that ranged from *not at all* (1) to *very much so* (5). The formulation and content of the measure were reviewed by an expert panel, which consisted of licensed coaches who used to be members of the national wheelchair basketball team themselves.

#### Sport-Specific Self-Efficacy and Competitive Anxiety

To assess sport-specific self-efficacy^1^ and the two dimensions of competitive anxiety (i.e., somatic arousal and cognitive worry), the 12-item *Kurzfragebogen Wettkampf-Angst Inventar – State* (WAI-S) was used. This questionnaire was developed by Brand et al. (2009) and was modeled after the widely used Competitive State Anxiety Inventory-2R (cf. Martens et al., 1990; Cox et al., 2003). Respondents were instructed to indicate on a four-point Likert scale, ranging from *not at all* (1) to *very much so* (4), how they felt 30-40 min before the start of the game (Martens et al., 1990; Cox et al., 2003). The *sport-specific self-efficacy* subscale aims to capture feelings of being able to perform well (e.g., “Right now, at this moment, I am confident about performing well”). The *cognitive worry* subscale aims to capture worries about performance (e.g., “Right now, at this moment, I am concerned that others will be disappointed with my performance”), whereas the *somatic arousal* subscale is concerned with bodily signs of stress (e.g., “Right now, at this moment, my heart is racing”). An index was created for each four-item subscale by averaging each participant’s scores, which resulted in scores ranging from 1 (low intensity) to 4 (high intensity). Brand et al. (2009) conducted an extensive assessment of the WAI-S factorial structure and the internal consistency of its subscales and they report values of Cronbach’s α coefficient ranging from 0.74 to 0.82. In the current sample, Cronbach’s α was 0.71 for sport-specific self-efficacy, 0.65 for cognitive worry, and 0.63 for somatic arousal.

#### Game Performance

Two performance indicators were used to measure game performance: (1) Coaches were asked to rate the game performance of their players on a 10-point Likert scale, ranging from *very bad* (1) to *very good* (10). These performance evaluations were collected immediately after the target game, that is, the game that the participants played after filling out the WAI-S questionnaire. (2) The number of points scored in the target game was adopted as a second performance indicator. To statistically control for players’ average level, their pre-game average, that is, the average number of points scored per game over all the games prior to the target game, was included as a covariate in the subsequent analyses. The data on these scored points were gathered from the game sheets of the specific games that were available online at the *Ergebnisdienst*, which is part of the website of the Fachbereich Rollstuhlbasketball of the Deutschen Rollstuhl Sportverband.

### Data Analysis

**FIGURE 2:**
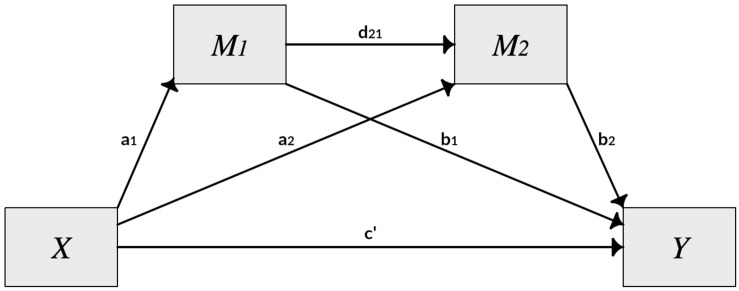
Statistical diagram of model 6 of the PROCESS macro, based on Hayes (2018).

In order to test the research model displayed in Figure 1, Hayes (2018) regression-based PROCESS macro for SPSS was used. Model 6 of this macro allows for carrying out serial multiple mediator analyses. The statistical diagram in Figure 2 shows that in a model with two mediators, the total effect of *X* on *Y* can be partitioned into a network of direct and indirect components. One “pathway” runs directly between *X* and *Y* and represents the direct effect. The remaining effect is indirect and runs through another three separate pathways. Based on ordinary least squares (OLS) regression, PROCESS provides estimates of parameters for all the direct components within the pathways, including the direct effect of *X* on *Y*. In addition to these parameters, PROCESS generates bootstrap confidence intervals to estimate the indirect effect(s) of *X* on *Y*.

The PROCESS analysis is restricted to only one dependent variable at a time. Therefore, separate analyses were to be carried out for each of the outcome variables in the model. To simulate that the two dependent variables were part of the same procedure, a seed of 11048 was specified for the random number generator of SPSS prior to running PROCESS. This ensured that the same standard errors and confidence intervals would be generated each time the program was run, with the *Y* variable as the only changing factor. An additional advantage of this fixed seed is that it enhances the replicability of the research (Hayes, 2018).

To check the common assumptions for regression-based procedures, a so-called “fake regression” of all the variables in the model against a chi-squared random variable with seven degrees of freedom was conducted beforehand. The resulting scatterplot of residuals versus predicted values gave reason to question the assumption of equal variances. Therefore, the HC3 heteroscedasticity-consistent standard error estimator was adopted (Hayes and Cai, 2007). A *p-*value of 0.05 was used to determine the statistical significance of the parameters for *direct* effects between the different pairs of antecedent and consequent variables captured in the model. For the *indirect* effects, the confidence level of the bootstrap confidence intervals was set to 95% and 10,000 bootstrap samples were used. If zero occurs between the lower limit (LBCI) and the upper limit (UBCI) of the interval, then we can conclude (with 95% confidence) that the true indirect effect is zero (that is, no mediation).

## Results

### Descriptive Data

Table 1 shows the demographic characteristics of the study sample. Of the 141 wheelchair basketball athletes who participated in the study, the majority (82.3%) were male. Furthermore, 93.6% were older than 18 years of age, and 93.6% had more than two seasons of experience. For the latter two variables, only the dichotomized data is available. As discussed, the age of 18 and 2 years of experience are cut-off levels for potential bonus points added to the classification system. The classifications of the participants ranged from 1.0 to 4.5 (*M* = 2.99, *SD* = 1.34). All eight classes were represented in the sample, but 39.0% of the participants were classified 4.0 or 4.5. In addition to these demographic characteristics, Table 2 provides summary statistics of the scoring performance of the participating athletes in the lowpoint (1.0–1.5), midpoint (2.0–3.5), and highpoint (4.0–4.5) classes.

**TABLE 2:** Summary statistics of points scored for lowpoint, midpoint, and highpoint athletes.

Functional class group	Mean score	*SE*	Minimum	Maximum
Low (1.0–1.5)	2.00	0.55	0	12
Mid (2.0–3.5)	6.22	0.94	0	30
High (4.0–4.5)	8.42	1.15	0	29

### Preliminary Analyses

The correlations reported in Table 3 show that classification was not related to any of the psychological variables. The statistically significant correlation between classification and sex indicates that 64.0% of the females versus 33.6% of the males had a classification of 4.0/4.5. Although classification and perceived competitive demands were unrelated, the link between sex and perceived competitive demands implies that higher classified athletes (i.e., females) were more likely to perceive competitive demands as higher [*M*_*female*_ = 2.48, *SD* = 0.65 versus *M*_*male*_ = 2.05, *SD* = 0.81, *t*(139) = −2.47, *p* = 0.015].

**TABLE 3:** Correlations, means, and standard deviations.

		*M*	*SD*	1	2	3	4	5	6	7	8	9	10	11
1	Competition level^1^	2.83	0.94	–										
2	Sex	0.18	0.38	0.06	–									
3	Age	0.94	0.25	0.08	0.12	–								
4	Experience	0.94	0.25	0.01	0.12	0.05	–							
5	Classification	2.99	1.34	–0.01	0.18*	–0.06	0.02	–						
6	Perceived demands	2.13	0.80	−0.17*	0.21*	–0.03	–0.03	–0.01	–					
7	Self-efficacy	2.92	0.54	0.18*	–0.10	–0.09	0.11	0.05	−0.35***	–				
8	Cognitive worry	1.64	0.48	–0.01	0.13	0.06	–0.11	0.08	0.20*	−0.48***	–			
9	Somatic arousal	1.45	0.45	0.02	–0.04	0.07	–0.09	–0.09	0.08	−0.17*	0.32***	–		
10	Points scored	6.12	7.36	0.12	–0.13	–0.02	0.16	0.30***	−0.37***	0.25**	–0.07	–0.06	−	
11	Coach evaluation	6.63	1.39	0.02	0.14	–0.11	0.12	0.00	−0.17*	0.15	0.01	0.09	0.20*	–
12	Pre-game average	6.36	6.13	0.08	−0.17*	0.00	0.12	0.39***	−0.39***	0.21*	–0.12	–0.05	0.80***	0.07

^1^For the purpose of convenience, the “Competition level” variable was reverse coded with the lowest value representing the lowest league (Landesliga) and the highest value representing the highest league (1e Bundesliga). *p < 0.05; **p < 0.01; ***p < 0.001 (two-tailed). Sex: 0 = male, 1 = female. Age: 0 = ≤ 18 years, 1 = > 18 years. Experience: 0 = < 2 seasons, 1 = ≥ 2 seasons (bonus points).

Furthermore, Table 3 shows that classification was associated with both pre-game average and number of points scored in the target game. These correlations between classification and objective measures of game performance indicate that athletes with higher classifications contributed more to their team’s score.

In line with our research model (see Figure 1), statistically significant correlations were observed between perceived competitive demands and sport-specific self-efficacy as well as between these two variables and the dependent variable of cognitive worry. Somatic arousal appeared to be unrelated to perceived competitive demands and was therefore not included as a dependent variable in subsequent analyses.

Although the two indicators of game performance were significantly related to each other, only the number of points scored in the target game was related to both perceived competitive demands and sport-specific self-efficacy. Therefore, the coach evaluation was also excluded as a dependent variable in subsequent analyses.

### Model Testing

The serial multiple mediator analyses were carried out for cognitive worry (*Y*_1_) and points scored (*Y*_2_). In both analyses, classification was treated as the independent variable (*X*), whereas perceived competitive demands served as the first mediator (*M*_1_), and sport-specific self-efficacy served as the second mediator (*M*_2_). Because perceived competitive demands and sport-specific self-efficacy were related to competition level and sex, these latter variables were included as covariates (*C*_1_-*C*_2_). As discussed in Section “Materials and Methods,” pre-game average was also included as a covariate (*C*_3_). Seven participants were excluded from the analyses because no pre-game average was available (*n* = 134).

In line with the correlations (see Table 3), the results of the serial mediation analyses presented in Table 4 show no significant direct effects of classification on perceived competitive demands, self-efficacy, or cognitive worry. When statistically controlling for pre-game average, there was also no longer a direct effect of classification on points scored. The same conclusions were reached when separate analyses were performed for male and female athletes and for different sets of functional classes (low: 1.0–1.5; mid: 2.0–3.5; and high: 4.0–4.5). Consequently, as indicated by the bootstrap confidence intervals in Table 4 (i.e., zero lies within the interval range), none of the indirect effects of classification on cognitive worry or points scored were statistically significant (whether via perceived competitive demands, sport-specific self-efficacy, or both).

**TABLE 4:** Regression table for serial mediation analysis with cognitive worry, and game performance as dependent variables.

Antecedent	Consequent															
	Perceived demands (*M*_1_)				Self-efficacy (*M*_2_)				Cognitive worry (*Y*_1_)				Points scored (*Y*_2_)			
	*B*	*SE*	*t*	*p*	*B*	*SE*	*t*	*p*	*B*	*SE*	*t*	*p*	*B*	*SE*	*t*	*p*
Classification (*X*)	0.05	0.06	0.95	0.343	0.02	0.04	0.45	0.653	0.04	0.04	1.26	0.209	–0.12	0.32	–0.37	0.709
Perceived demands (*M*_1_)	–	–	–	–	–0.20	0.07	–2.85	0.005	0.02	0.06	0.26	0.792	–0.31	0.60	–0.52	0.607
Self-efficacy (*M*_2_)	–	–	–	–	–	–	–	–	–0.42	0.07	–6.16	<0.001	1.02	0.81	1.26	0.209
Competition level (*C*_1_)	–0.13	0.05	–2.53	0.013	0.08	0.06	1.36	0.176	0.04	0.04	0.89	0.375	0.27	0.55	0.49	0.625
Sex (*C*_2_)	0.35	0.16	2.16	0.033	–0.09	0.14	–0.68	0.501	0.02	0.10	0.17	0.863	0.49	0.84	0.58	0.562
Pre-game average (*C*_3_)	–0.05	0.01	–4.19	<0.001	0.01	0.01	0.54	0.587	–0.01	0.01	–0.72	0.475	0.96	0.11	8.38	<0.001
Constant	2.57	0.24	10.69	<0.001	3.04	0.27	11.28	<0.001	2.63	0.32	8.26	<0.001	–2.62	3.23	–0.81	0.419
	*R*^2^ = 0.21				*R*^2^ = 0.15				*R*^2^ = 0.26				*R*^2^ = 0.65			
	*F*(4,129) = 10.61, *p* < 0.001				*F*(5,128) = 4.78, *p* < 0.001				*F*(6,127) = 8.85, *p* < 0.001				*F*(6,127) = 26.62, *p* < 0.001			

									**Effect**	***SE***	**LBCI**	**UBCI**	**Effect**	***SE***	**LBCI**	**UBCI**

Indirect effect 1 (*X → M_1_ → Y*)									0.001	0.004	–0.008	0.010	–0.017	0.049	–0.141	0.061
Indirect effect 2 (*X → M_2_ → Y*)									–0.008	0.017	–0.044	0.026	0.019	0.054	–0.081	0.145
Indirect effect 3 (*X → M_1_ → M_2_ → Y*)									0.005	0.005	–0.005	0.015	–0.011	0.016	–0.049	0.018

*LBCI = lower 95% Bootstrap confidence interval; UBCI = upper 95% Bootstrap confidence interval. Sex: 0 = male, 1 = female.*

### Reduced Model

**FIGURE 3:**
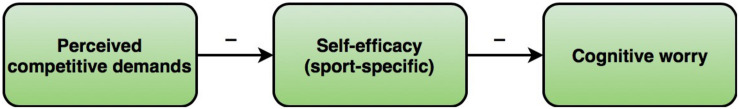
Reduced model based on research findings, with plus and minus signaling the direction of hypothesized relationships.

Due to the significant zero-order correlations that were found between perceived competitive demands, self-efficacy, and the dependent variable cognitive worry (see Table 3), we subsequently tested a reduced model (i.e., without functional classification), namely, the indirect relationship between athletes’ appraisal of competitive demands and cognitive worry through the mediating variable of sport-specific self-efficacy (see Figure 3).

This simple mediation analysis was conducted using Model 4 of the PROCESS macro. The athlete’s perception of competitive demands was entered as the independent variable (*X*), self-efficacy served as the mediator variable (*M*), and cognitive worry (*Y*) was entered as the dependent variable. We included only competition level as a covariate because sex was unrelated to sport-specific self-efficacy and cognitive worry (see Table 4). The full sample of participants was included in the analysis (*n* = 141).

The results, presented in Table 5, suggest that athletes who rated the competition demands higher felt less self-efficacious, and accordingly, reported higher intensities of cognitive worry. The statistical significance of this indirect effect was confirmed by the bootstrap confidence interval, which did not include zero.

**TABLE 5:** Regression table for simple mediation analysis with cognitive worry as dependent variable.

Antecedent	Consequent							
	Self-efficacy (*M*)				Cognitive worry (*Y*)			
	*B*	*SE*	*t*	*p*	*B*	*SE*	*t*	*p*
Perceived demands (*X*)	–0.22	0.06	–3.90	<0.001	0.03	0.05	0.56	0.579
Self-efficacy (*M*)	–	–	–	–	–0.42	0.07	–6.38	<0.001
Competition level (*C*)	0.07	0.05	1.31	0.191	0.04	0.04	0.99	0.325
Constant	3.18	0.21	15.10	<0.001	2.71	0.28	9.62	<0.001
	*R*^2^ = 0.13				*R*^2^ = 0.24			
	*F*(2,138) = 9.79, *p* < 0.001				*F*(3,137) = 16.04, *p* < 0.001			

					**Effect**	***SE***	**LBCI**	**UBCI**

Indirect effect (*X → M → Y*)					0.093	0.027	0.044	0.152

*LBCI = lower 95% Bootstrap confidence interval; UBCI = upper 95% Bootstrap confidence interval.*

## Discussion

The primary objective of the present study was to explore the role of functional classification as a competitive stressor for wheelchair basketball athletes. Specifically, we theorized that the ordinal scale of the system may increase athletes’ concern about performing in accordance with one’s functional class. To examine this, we tested a serial mediation model in which functional classification affects cognitive and somatic dimensions of competitive anxiety and game performance, through the sequence of perceived competitive demands and sport-specific self-efficacy (see Figure 1). Unexpectedly, the results provided no support for a positive relationship between functional classification and perceived competitive demands. Furthermore, we did not find direct or indirect effects of classification on sport-specific self-efficacy and the outcome variables cognitive worry, somatic arousal, and game performance. For coaches and other persons involved in mentally preparing wheelchair basketball athletes for games, these results are important because they suggest that concerns about performing in accordance with their functional class are not experienced differently by athletes from different functional classes. In other words, we found no empirical evidence for considering class-related differences when dealing with athletes’ (self-imposed or other-imposed) performance expectations.

The finding that functional classification and perceived competitive demands were unrelated may be explained by the observed positive correlations between functional classification and the objective game performance data (see Table 3). That is, higher classified athletes tend to contribute more to a team’s score (cf. Vanlandewijck et al., 2004; Mishin, 2017; Doi et al., 2018). These correlations suggest that performance differences between functional classes are quite robust, and accordingly, validate the proportionality of the system. Hence, an athlete’s performance expectations and performance self-evaluation—which may lead to performance concerns—may be primarily based upon social comparisons *within* the athlete’s own functional class, i.e., with similar others in terms of classification (cf. Festinger, 1954; Wheeler et al., 1982). When evaluating their game performance, midpoint athletes with a 2.5 classification, for example, may primarily rely on social comparison information from 2.0 to 3.0 ranged athletes. Similarly, highpoint 4.0 athletes may evaluate their game performance relative to that of athletes around 4.0. Such a tendency would equalize athletes’ perceived competitive demands across classes.

In our sample, female wheelchair basketball athletes—64.0% of whom belonged to the highpoint classes 4.0/4.5—reported higher competitive demands, which might be interpreted as indirect evidence of a relationship between classification and perceived demands. However, instead of classification, alternative explanations for the higher perceived competitive demands among female athletes may be their tendency to focus more on the risk of failure rather than on achieving success and their higher susceptibility to environmental pressure (Silva Rocha and de Lima Osório, 2018). Sex differences are often explained by differences in (sports) socialization. For example, male athletes may have developed a more dominant competitive orientation and have a stronger tendency to prove and protect their competence because they engaged more in competitive games and sports when they were young. Accordingly, male athletes may feel more comfortable in a competitive sports context (Jones et al., 1991; Feltz et al., 2008; cf. Van Yperen et al., 2014; Butler and Hasenfratz, 2017). Campbell and Jones (1997) also refer to this socialization effect when discussing the decrease in self-confidence immediately prior to competition among female athletes in various adaptive sports, which might be an explanation for the higher perceived demands in a competitive context among female athletes.

Furthermore, the absence of a classification effect on athletes’ game performance may be attributed to athletes’ consistency in game performance. As shown in Table 3, the correlation between athletes’ pre-game average and points scored in the target game was 0.80 (i.e., 64% explained variance), which is in line with the general finding that past performance is the best predictor of future performance (e.g., Wernimont and Campbell, 1968; Ouellette and Wood, 1998; Niessen et al., 2016). A similar consistency was found by Katartzi et al. (2007), who examined the passing performance of wheelchair basketball athletes. Also note that statistically, the very high correlation between past (pre-game average) and current performance (points scored) leaves very little variance to be explained by additional (psychological) variables. Indeed, excluding pre-game average as a covariate revealed a direct effect of classification on points scored (*B* = 1.74, *SE* = 0.42, *p* < 0.001; cf. Table 3). This finding underlines what is said above about the robustness of the functional classification system and that higher classified athletes quite consistently contribute more to a team’s score (Vanlandewijck et al., 2004; Mishin, 2017; Doi et al., 2018).

Regardless of classification, in the present research, we found support for a reduced model, namely, the mediating effect of sport-specific self-efficacy on the relationship between athletes’ appraisal of competitive demands and the amount of cognitive worry they experience prior to competition (see Figure 3). The negative effect of self-efficacy on cognitive worry, indicating that self-efficacious athletes are less likely to experience cognitive manifestations of competitive anxiety, is well-established in research among able-bodied athletes (Feltz et al., 2008). Our results provide empirical evidence that the relationship between self-efficacy and cognitive worry in wheelchair basketball athletes follows a similar pattern. Together, these findings may have practical value for coaches and applied sports psychologists in wheelchair basketball because they highlight the importance of both managing athletes’ appraisal of competitive demands and enhancing their feelings of self-efficacy.

In addressing athletes’ perception of competitive demands, one strategy is to build team cohesion, primarily by enhancing commitment to a common task, but also by developing interpersonal attraction and pride in the group itself (Mullen and Copper, 1994). For example, Wolf et al. (2015) found that athletes who perceived their team as more cohesive felt more accepted by their team members, experienced greater social support, and accordingly, fewer competitive demands. Reducing athletes’ perception of competitive demands through team cohesion interventions may be particularly effective at the level of national wheelchair basketball teams. Players from these teams come from all over the country and typically have infrequent (offline) communication, and therefore it requires more effort to establish a firm sense of team cohesion (Martin, 2018).

The empirical support for the reduced model also illuminates the potential effectiveness of self-efficacy-based interventions to reduce athletes’ (cognitive) competitive anxiety. For example, in Koestner’s et al. (2006) self-efficacy boosting training, participants were instructed: (a) to formulate a goal that had already been achieved similar to the goal currently being pursued, (b) to think of someone similar to themselves who had already attained the goal being pursued, and (c) to think of an individual who could offer support in attaining the goal. These instructions are rooted in three of the four major sources of self-efficacy beliefs, namely, mastery experiences, vicarious experiences, and verbal persuasion (Bandura, 1997; Short and Ross-Stewart, 2009). By targeting self-efficacy at the source, coaches and applied sports psychologists may help athletes to become more self-efficacious and, accordingly, less worried about their future game performance.

When interpreting the current findings, a number of limitations should be considered. First, the empirical support for our reduced model is based solely on self-reported data. Self-presentation behavior among the participants may have been elicited by the request to write their names on the questionnaire. By emphasizing that all information provided by the participants would be treated with confidentiality, however, we have tried to minimize this potential bias. Second, perceived competitive demands was measured by one single item. This was because we had very limited time and questionnaire space, and we had to reduce the burden on participants. For our research purposes, we feel that this straightforward item was useful (cf. Wanous et al., 1997; Drolet and Morrison, 2001; Davey et al., 2007). Third, the two performance indicators used to assess game performance did not reflect the multifaceted nature of wheelchair basketball performance. However, the coaches’ evaluations of their players’ individual game performance may be considered as an assessment that covers all technical, tactical, physical, and mental aspects of the game. This measure was indeed only moderately correlated with the number of points scored in the target game (see Table 3). Nevertheless, also with this performance index, we did find empirical support for our research model.

## Conclusion

Functional classification is essential in adaptive sports, and it has been the subject of many studies. Nonetheless, the role of classification as a potential stressor has been largely ignored. In the present study, we aimed to explore the anecdotal relationship between classification and athletes’ concern about “performing in accordance with one’s class,” by examining the impact of functional classification on competitive anxiety and game performance among wheelchair basketball athletes. Although no direct or indirect effects of classification were found on perceived competitive demands, sport-specific self-efficacy, or competitive anxiety, the results do highlight some interesting points. First, we found positive correlations between functional classification and athletes’ contribution to their team’s score (pre-game average and points scored), which is in line with research supporting the proportionality of the functional classification system in wheelchair basketball and underlines the robustness of the system. Second, the proportionality of the functional classification system appeared not to be associated with differences in the appraisal of competitive demands between the functional classes. This indicates that when athletes are mentally preparing for their games, class-related group differences may not be relevant with regard to managing their perception of competitive demands, and accordingly, their sport-specific self-efficacy and cognitive competitive anxiety. Third, and related, we demonstrated a negative relationship between perceived competitive demands and cognitive competitive anxiety through sport-specific self-efficacy. These findings suggest that, regardless of classification, athletes’ cognitive competitive anxiety may be reduced by interventions focused on reducing athletes’ perception of competitive demands (e.g., through developing team cohesion) and enhancing their sport-specific sense of self-efficacy.

## Data Availability Statement

The raw data supporting the conclusions of this article will be made available by the authors, without undue reservation.

## Ethics Statement

The studies involving human participants were reviewed and approved by the Ethical Committee of Psychology (ECP) of the University of Groningen, Netherlands. The participants provided their informed consent to participate in this study. Written informed consent from the participants’ legal guardian/next of kin was not required to participate in this study in accordance with the national legislation and the institutional requirements.

## Author Contributions

NB developed the theoretical framework, conceived the study, analyzed the data, and wrote the manuscript. NV conceived the study, guided the analyses, and provided critical feedback on the drafts. Both authors contributed to the article and approved the submitted version.

## Conflict of Interest

The authors declare that the research was conducted in the absence of any commercial or financial relationships that could be construed as a potential conflict of interest.

## Footnotes

1 The German name for the subscale of sport-specific self-efficacy is *Zuverzicht*, which is commonly translated as “self-confidence.” The latter is also the name of the corresponding CSAI-2 subscale. In both cases, the subscale items address the participant’s situation- and skill-specific sense of competence (i.e., self-efficacy), which is emphasized by the specific context in which the questionnaire is administered.
